# Agricultural intensification, priming for persistence and the emergence of Nipah virus: a lethal bat-borne zoonosis

**DOI:** 10.1098/rsif.2011.0223

**Published:** 2011-06-01

**Authors:** Juliet R. C. Pulliam, Jonathan H. Epstein, Jonathan Dushoff, Sohayati A. Rahman, Michel Bunning, Aziz A. Jamaluddin, Alex D. Hyatt, Hume E. Field, Andrew P. Dobson, Peter Daszak

**Affiliations:** 1 Department of Ecology and Evolutionary Biology, Princeton University, Princeton, NJ 08544, USA; 2 Fogarty International Center, National Institutes of Health, Bethesda, MD 20892, USA; 3 EcoHealth Alliance (formerly Wildlife Trust), 460 West 34th Street, 17th Floor, New York, NY 10001, USA; 4 Department of Veterinary Services, Veterinary Research Institute, Ipoh, Perak, Malaysia; 5 Universiti Putra Malaysia, Serdang, Selangor, Malaysia; 6 59th, Medical Wing, Lackland Air Force Base, USAF, San Antonio, TX 78201, USA; 7 Department of Veterinary Services, Ministry of Agriculture, Putrajaya, Malaysia; 8 Australian Animal Health Laboratory, Commonwealth Scientific and Industrial Research Organization (CSIRO), Geelong, Victoria, Australia; 9 Biosecurity Sciences Laboratory, Department of Employment, Economic Development and Innovation, Brisbane, Queensland, Australia

**Keywords:** Nipah virus, epidemic enhancement, infectious disease dynamics, emerging pathogens, zoonosis, flying fox

## Abstract

Emerging zoonoses threaten global health, yet the processes by which they emerge are complex and poorly understood. Nipah virus (NiV) is an important threat owing to its broad host and geographical range, high case fatality, potential for human-to-human transmission and lack of effective prevention or therapies. Here, we investigate the origin of the first identified outbreak of NiV encephalitis in Malaysia and Singapore. We analyse data on livestock production from the index site (a commercial pig farm in Malaysia) prior to and during the outbreak, on Malaysian agricultural production, and from surveys of NiV's wildlife reservoir (flying foxes). Our analyses suggest that repeated introduction of NiV from wildlife changed infection dynamics in pigs. Initial viral introduction produced an explosive epizootic that drove itself to extinction but primed the population for enzootic persistence upon reintroduction of the virus. The resultant within-farm persistence permitted regional spread and increased the number of human infections. This study refutes an earlier hypothesis that anomalous El Niño Southern Oscillation-related climatic conditions drove emergence and suggests that priming for persistence drove the emergence of a novel zoonotic pathogen. Thus, we provide empirical evidence for a causative mechanism previously proposed as a precursor to widespread infection with H5N1 avian influenza and other emerging pathogens.

## 1. Introduction

Preventing and controlling emerging zoonoses require identification of the processes that drive cross-species pathogen transmission [1]. Agricultural intensification has been proposed as a major underlying cause of pathogen emergence from wildlife and domestic animal populations into human populations [2,3]; however, the precise mechanisms by which this occurs have rarely been demonstrated. Specific agricultural practices may increase frequency of cross-species pathogen transmission, setting the stage for persistence of new pathogens to occur. Pathogen introduction into a partially immune population—such as a population where some individuals are vaccinated or were previously infected—can result in longer and potentially larger epidemics than introduction into a naive population [4]. We refer to this phenomenon as immunity-based, population-level ‘priming’ for persistence. The potential of such priming to drive zoonotic emergence has been demonstrated theoretically in the general case [4], as a possible precursor to measles emergence [5] and as a mechanism that could facilitate widespread emergence of H5N1 avian influenza in poultry [6]. Here, we present evidence that this process is not only theoretically possible but is likely to have played a key role in the first known outbreak of Nipah virus (NiV) encephalitis, and therefore for the emergence of a lethal zoonosis.

### 1.1. Background

NiV is a paramyxovirus that emerged in people in Malaysia in 1998 [7–9]. Serology, virus isolation and polymerase chain reaction detection indicate that NiV is maintained in *Pteropus* spp. fruit bats (flying foxes), including *P. vampyrus* and *P. hypomelanus* in peninsular Malaysia [10–12]. Transmission from flying foxes to pigs is thought to occur via saliva on fomites (discarded fruit pulp) or via faecal or urine contamination of pigsties [11]. Pigs act as amplifier hosts, enabling infection of humans via droplet transmission during respiratory infections [13]. The risk of direct transmission from bats to humans in Malaysia is believed to be low [14].

**Figure 1.**
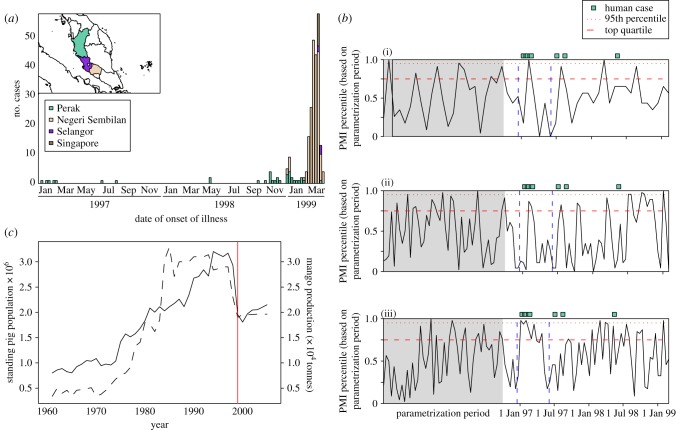
(*a*) Epidemiological data from the entire outbreak region, (*b*) the index farm and (*c*) evidence of agricultural intensification. (*a*) Symptomatic Nipah virus cases in Malaysia and Singapore, January 1997–April 1999; data are from the Center for Disease Control and Prevention's *Morbidity and Mortality Weekly Reports* [7,8], records from the Department of Veterinary Services and the Ministry of Health, Malaysia [16]. Further information on case numbers can be found in the electronic supplementary material. (*b*) Detection of Nipah virus-induced pre-weaning mortality in the three breeding sections on the index farm. To investigate the timing of Nipah-induced mortality in the three breeding sections, we define a piglet mortality index (PMI) that compares the observed mortality for each litter with expected mortality (based on the number of piglets born and the time from farrow to wean). Here we present average PMI values through time, shown as quantiles of the values observed during the parametrization period; raw litter-level PMI values are shown in the electronic supplementary material. The presence of Nipah virus is suggested when pre-weaning mortality of piglets is significantly greater than expected, and we infer the presence of Nipah virus when significant excess mortality is observed in multiple litters during the same time period or coincident with human cases. The binned PMI values show that significant mortality peaks occurred in the Breeding Nucleus and Breeding section 2 during the time period of the first cluster of human cases in early 1997; individual litters in Breeding section 1 also showed elevated mortality during this time period (electronic supplementary material). Piglet mortality then returned to baseline levels until approximately July 1997, corresponding to the time of the next observed human cases, around which time the virus is likely to have been reintroduced from the flying fox reservoir. Model output analysed in the same manner as the piglet mortality data from the index farm is shown in the electronic supplementary material. (i) Breeding Nucleus; (ii) Breeding section 1; (iii) Breeding section 2. (*c*) Commercial production of mangoes and domestic pigs in Malaysia, 1961–2005. The drop in both mango (dashed lines) and pig production (solid line) between 1998 and 1999 is because of the depopulation and closing of pig farms in areas infected with Nipah virus. Mangoes on these farms were not harvested and many of the orchards have since been abandoned or converted to other crops. Data are from the Food and Agricultural Organization's Agricultural Production database.

Between September 1998 and April 1999, NiV caused 246 reported cases of febrile encephalitis in humans in peninsular Malaysia and Singapore [7,8] and an epidemic of respiratory and neurological disease in commercially farmed pigs [9,15]. Initial human cases were seen in Tambun village and surrounding areas in Perak State, followed by a large epidemic in the southern states of Negeri Sembilan and Selangor and several cases in Singapore (figure 1*a*). The majority of these cases occurred in pig farmers and abattoir workers [17]. During the course of the outbreak investigation, nine cases of NiV encephalitis were retrospectively diagnosed in the Tambun area with onset dates between January 1997 and May 1998, five of which were associated with the index farm of the 1998–1999 outbreak in Perak (figure 1*a*). Six of the nine cases are considered confirmed cases on the basis of serological tests performed in 1999 [16]; the remaining three are probable cases.

Several mechanisms have been previously proposed for the appearance of NiV in pig and human populations in 1998. One frequently cited explanation is that flying foxes harbouring NiV were new to the region where transmission to pigs occurred, driven there by climatic anomalies. In late 1997, an El Niño Southern Oscillation-related drought and burning of forested land in Sumatra and Kalimantan (Indonesia) created an atmospheric haze across Sumatra and peninsular Malaysia [18]. One of us (K.B.C., see group author list) hypothesized that this atmospheric haze caused atypical flying fox immigration from Sumatra across the Straits of Malacca into peninsular Malaysia and northward towards Perak [18]. This hypothesized movement of flying foxes into an area with large pig farms potentially outside the species' normal range was proposed to have precipitated transmission of the virus from bats to pigs. However, a retrospective diagnosis of human NiV encephalitis cases on the index farm in early 1997 indicates that bat-to-pig transmission did not result from these specific environmental conditions. In particular, seven of these cases occurred prior to the rise in airborne particulate matter that is diagnostic of the haze event, which peaked in September 1997 [18]. The recognition of cases prior to the haze event refutes the hypothesis that this event drove initial cross-species transmission (table 1). Furthermore, at the time of the major outbreak in pigs and humans, NiV antibodies were found to be widespread in flying fox colonies within peninsular Malaysia [12], suggesting that the outbreak was not the result of a (recent) point source introduction of the virus into the bat population, as previously suggested [18].

**Table 1.** Proposed drivers of emergence. Hill outlined nine criteria to be used to assess causality for epidemiological outcomes with complex origins [19]. A useful discussion of the implementation of these criteria in disease ecology is provided by Plowright *et al*. [20]. This table is intended to assess the causal role of three factors that have been proposed as drivers of Nipah virus emergence in Malaysia. Because we are assessing causality in the context of a specific event, three of the nine original criteria are considered inapplicable (strength of association, consistency and biological gradient). n.a., not applicable.

proposed driver	relevant outcome	Hill's criterion^a^	support for causality	explanation	additional information	assessment
agricultural intensification (i.e. increasing production and dual use of agricultural land)	phase I emergence	plausibility	yes	increased linkage between fruit crops and livestock is a plausible origin for the pathway for transmission from bats to pigs	figure 1*c*, electronic supplementary material, figure S5*a* and [18]	driver of phase I emergence: created pathway for transmission from flying foxes to pigs
		coherence	yes	explanation is consistent with the timing of fruit production on the index farm	timeline of phase I emergence in electronic supplementary material	
		experimental evidence	yes	no human cases have occurred in Malaysia since policies were put in place regulating the minimum distance between fruit trees and pigsties		
		analogy	yes	situation is similar to intense cultivation of date palm sap that provides a pathway for transmission from bats to people in Bangladesh	[21]	
		specificity	yes	there is no evidence of spillover from bats in areas where dual-use agriculture was not practised		
		**temporality**	**yes**	intensification occurred over several decades prior to emergence. On the index farm, mango trees adjacent to pig sties matured in 1987	figure 1*c*, timeline of phase I emergence in electronic supplementary material	
priming for persistence	phase II emergence	plausibility	yes	repeated introduction of the virus appears to have been necessary and sufficient for enzootic circulation (persistence) of the virus on the index farm. Sale of pigs from this farm to surrounding areas would have spread infection, and movement of infected pigs to the south would have resulted in phase II emergence	figures 1*b*, 2, 3; [22]	driver of phase II emergence: created conditions for persistence in a commercial pig population
		coherence	yes	explanation is consistent with prior accounts of the outbreak and all known data		
		experimental evidence	n.a.			
		analogy	yes	mechanism has been shown to be theoretically possible in a wide variety of systems and proposed as a possible precursor to widespread infection with H5N1 avian influenza	[4,6]	
		specificity	yes	the initial introduction was insufficient to produce the second phase of emergence	figure 1*a*	
		**temporality**	**yes**	putative initial introduction and reintroduction dates precede the second phase of emergence	figure 1*b*	
ENSO and haze event	phase I emergence	plausibility	yes	anomalous climatic conditions could affect flying fox movement and produce stressful conditions that increase viral shedding	[18]	not a driver of emergence
		coherence	no	human cases prior to the haze event violate the logically necessary criterion of temporality	figure 1*a*; [18]	
		experimental evidence	n.a.			
		analogy	yes	climate change has been implicated in emergence of other zoonotic infections		
		specificity	no	a similar haze event occurred in 2006 but did not result in human cases		
		**temporality**	**no**	the haze event occurred after the initial human cases	figure 1*a*; [18]	

^a^Temporality is the only criterion that is logically necessary (in bold).

### 1.2. Study rationale

Since its discovery in 1999, NiV has been identified as the cause of human neurological and respiratory disease in India and Bangladesh [23,24], and molecular evidence suggests a wide distribution of henipaviruses in the reservoir hosts [25–29]. Despite the continued threat that NiV and related viruses represent to global health, no detailed studies have combined data on pig populations, bat colonies and human cases to examine the mechanisms that drove the first known and largest outbreak. This paper describes an interdisciplinary approach that synthesizes all available data to improve and update our understanding of the process of NiV emergence in Malaysia. The study has three main branches, all of which build on earlier work.

First, Chua *et al*. [18] identified the presence of fruit trees on the index farm as a plausible link between flying foxes and pigs that could have precipitated introduction of the virus. We place this finding in a broader context by examining spatial and temporal patterns of agricultural production in peninsular Malaysia prior to and at the time of the outbreak. We also investigate, in detail, the history and status of fruit crop production on the index farm.

Second, our preliminary models suggested that repeated introduction could plausibly lead to persistence of the virus in a generic large pig population [30]. In this study, we use detailed information on management practices and production patterns at the index farm to parametrize models of this population. We use these models to examine whether repeated introduction was necessary and sufficient to allow the virus to persist on the farm. We then compare model predictions of piglet mortality patterns with data from production records to assess whether repeated introduction led to persistence of the virus in this population.

Third, Johara *et al*. [12] identified *Pteropus* spp. as likely reservoirs of NiV infection. Here we examine the distribution of *Pteropus* bats in peninsular Malaysia, present data on NiV serology from all known roosts and examine the opportunities for transmission of the virus from bats to pigs by placing these findings in the context of large-scale agricultural production patterns.

Finally, we bring together the three branches of the study to describe the events that led to NiV emergence in Malaysia and Singapore and discuss the causal nature of different contributing factors.

## 2. Results

### 2.1. Agricultural production patterns

#### 2.1.1. Large-scale patterns

Both pig and mango (*Mangifera indica*) production tripled in peninsular Malaysia between the early 1970s and the late 1990s (figure 1*c*). The intensification of production of pigs and mangoes was loosely correlated during this time period. The marked decline in both end-of-year standing pig population (SPP) and annual mango production between 1998 and 1999 indicates that this correlation may reflect widespread dual use of agricultural land to produce both pigs and mangoes, with the decline in commercial mango harvest reflecting the widespread abandonment and culling of infected pig farms that occurred in the first half of 1999 (following the discovery of NiV).

#### 2.1.2. Patterns on the index farm

Pigs were first brought to the land that became the index farm in 1970. By 1980, the SPP on the property had reached 30 000 (approx. the same size as at the time the farm was culled in 1999). Mangoes, jackfruit (*Artocarpus heteropyllus*) and durian (*Durio* sp.) were grown on the index farm, while other farms in the Tambun area grew primarily pomelos (*Citrus maxima*), which are not eaten by flying foxes. As Chua *et al*. [18] identified, several large mango trees were planted directly adjacent to pig enclosures. Approximately 400 mango trees were planted on the farm in 1983, and the trees began producing fruit in 1987. The location of these trees, in relation to the areas where pigs were kept, is shown in the electronic supplementary material, which also includes additional details of the timeline of pig and fruit production on the index farm.

### 2.2. Nipah virus on the index farm

#### 2.2.1. Dynamic models

**Figure 2.**
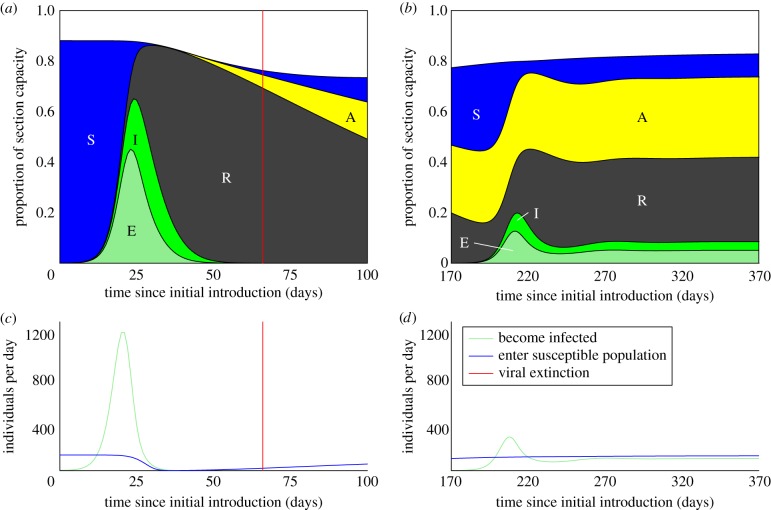
Deterministic model results illustrating Nipah virus dynamics in the growing section of the index farm. Individuals are characterized as belonging to one of five states: susceptible (S), immune—maternal antibodies (A), immune—recovered from infection (R), exposed (E) and infectious (I). The top panels illustrate the infection/immunity profile of the growing section following (*a*) initial introduction of the virus and (*b*) subsequent introduction. The population profile is normalized by the capacity of the growing section (see the electronic supplementary material). The qualitative difference in infection dynamics results primarily from the prevalence of maternal antibodies in the young pig population. (*c*,*d*) Following the initial introduction of the virus (*c*), the rate of replenishment of the susceptible population in the growing section (solid blue line) declines, as many individuals are immune, having been infected while in the breeding sections. The rate at which individuals are infected (green line) declines in consequence. When the virus is reintroduced (*d*), many individuals entering the growing section have maternal antibodies. Loss of maternal antibodies after entry into the growing section provides a source of susceptibles independent of the presence of infection (blue line), allowing the virus to persist. Infection dynamics are qualitatively similar for a wide range of transmission parameters. The results shown were produced using the following combination of transmission parameters: *ɛ* = 0.5, *σ* = 0.01 and *R*_0_ = 10. Analogous results for introduction into a vaccinated population are shown in the electronic supplementary material.

**Figure 3.**
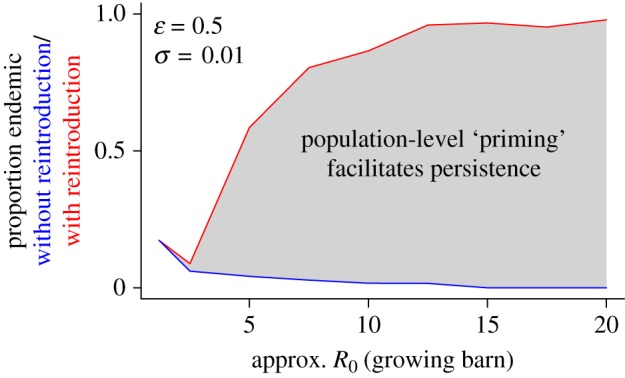
Results from an individual-based model of Nipah virus dynamics on the index farm: proportion of epidemics leading to enzootic circulation of Nipah virus on the index farm, with and without reintroduction of the virus. Simulations show that most epidemics produced by initial introduction of the virus drive themselves to extinction by depleting the susceptible population (blue line); however, a single reintroduction of the virus is most often sufficient to produce a subsequent epizootic that leads to persistent circulation. Here, we define persistence as circulation of the virus from the time of introduction (or reintroduction) until the farm was depopulated. Analogous results for a wide range of values for the unknown transmission parameters are shown in the electronic supplementary material.

We analysed pig production data from the index farm and developed an age-structured model of NiV dynamics within the farm population. Our model shows that multiple transmission events from bats to pigs are the best explanation for the observed pattern of human cases and pig mortality. As piglets aged in the index farm, they were moved from the breeding sections into the growing section, and finally into the finishing section for the last six weeks before they were sent to slaughter. When the virus was initially introduced into the pig population, individuals entering the growing section were immunologically naive. As the virus spread throughout the farm, the proportion of pigs entering this section with active immunity increased to the point where too few susceptibles remained for the chain of transmission to continue, and the virus could not be maintained within the farm population (figure 2*a,c*). The first, rapid epizootic of NiV in the pig population on the index farm thus would have produced highly localized human infections (figure 1*a*) and the virus most probably went extinct in the pig population at this time (figures 2*a,c* and 3).

Our models suggest that, when NiV was reintroduced to the farm, it circulated enzootically until the farm was depopulated in March 1999 (figures 2 and 3). This finding is consistent with the timing of human cases associated with the index farm and with the high antibody prevalence detected in sows and piglets in Breeding section 2 upon depopulation of the index farm [31]. The change in dynamics between initial and subsequent introductions of the virus on the index farm results from the presence of acquired immunity in the sow population and conferred immunity in the young pig population. The age structure within the growing section, along with its rapid turnover and large population size, allowed the virus to circulate enzootically. On reintroduction of the virus into the growing section, the population had a different immunological profile from the time of initial introduction. Many young pigs moving from the breeding sections into the growing section now carried maternally derived passive immunity rather than active immunity obtained from exposure to the virus (figure 2*b*). We estimate that maternal antibodies are lost at approximately 14 weeks of age (electronic supplementary material). Pigs born with maternal antibodies would therefore become susceptible to the virus roughly four weeks after entering the growing section, where they would remain for another six weeks. These dynamics produce a steady inflow of susceptible individuals, which is sufficient to maintain the virus over a period of 2 years or more (figure 2*b,d*), a period similar to that observed between the index case for NiV in Malaysia in 1997 and the onset of the large-scale outbreak. The population size and turnover rate of the finishing section suggest that it may also have permitted enzootic circulation of the virus (electronic supplementary material).

Stochastic simulations demonstrate that priming for persistence on the index farm is the most likely scenario consistent with pig and human epidemiology for *R*_0_ values >2.5 (up to *R*_0_ = 30, the highest value examined), and simulations with an *R*_0_ value in this range best reproduce the very high seroprevalence (>95% for sows and >90% for piglets) detected in Breeding section 2 at the time of depopulation [31] (electronic supplementary material).

#### 2.2.2. Data analysis

Although no direct prevalence data exist for NiV in the pig population on the index farm, the presence of the virus in the breeding sections can be inferred from production records kept by the farm's managers (electronic supplementary material) and from reported serological findings [31]. These data are consistent with the scenario predicted by the model and with the timing of human cases: there was a period of approximately five to six months between the first introduction of the virus into the pig population and subsequent, smaller peaks in piglet mortality (figure 1*b*), when the virus was probably reintroduced into the farm—probably from the flying fox reservoir, or possibly from other pig farms in the area. The virus appears to have spread into each of the three breeding sections between December 1996 and February 1997, with strong evidence of infection (unusually high piglet mortality) in at least two of the breeding sections (Breeding Nucleus and Breeding section 2) during January 1997, and significant mortality on a number of subsequent occasions, following an interim period in which piglet mortality returned to baseline levels (figure 1*b* and electronic supplementary material).

### 2.3. Bat surveys

#### 2.3.1. Bat distribution

**Figure 4.**
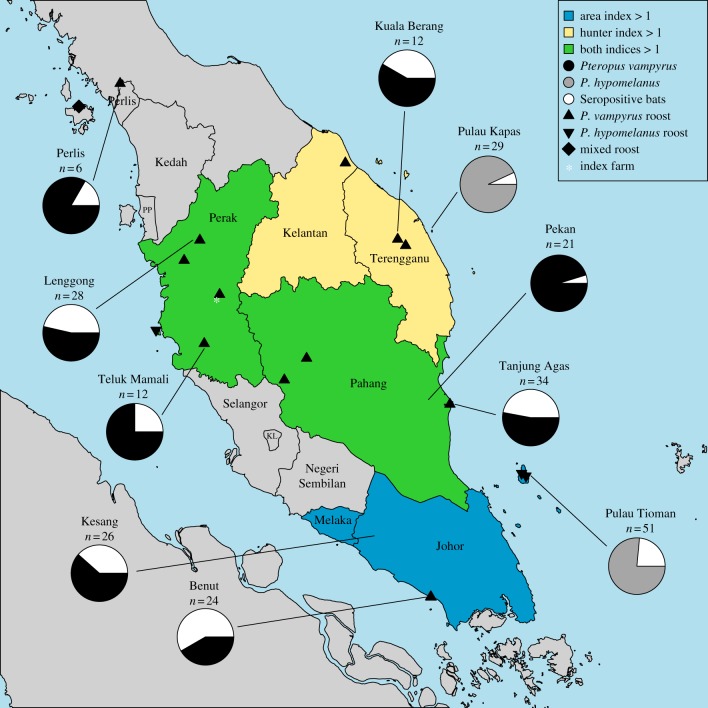
Nipah virus serology and flying fox distribution in peninsular Malaysia. The flying fox density in each state is expected to correlate with two indices that describe the hunting level within the state relative to a null expectation, as described in the electronic supplementary material. Hunting indices > 1 indicate a higher than expected hunting level and are expected to correlate with relatively high flying fox densities. Known flying fox roost locations tend to be located in states with relatively high hunting indices. Nipah virus serology results are given for all sampling sites throughout peninsular Malaysia; seroreactive bats (white) were found at all sampling sites. PP, Pulau Penang; KL, Kuala Lumpur.

We conducted a countrywide survey of the distribution and infection status of the two *Pteropus* species in peninsular Malaysia. We located 14 active *P. vampyrus* roost sites across peninsular Malaysia, four *P. hypomenlanus* roosts on islands surrounding the peninsula and one mixed roost near Langkawi Island off the northwest coast. We estimated the current distribution of *Pteropus* spp. bats in peninsular Malaysia using combined results of wildlife surveys and analysis of hunter licensing data (figure 4 and electronic supplementary material). The overall distribution of flying foxes in peninsular Malaysia is similar to that observed in a large-scale survey of flying fox populations conducted in 1999 [32], although there are fewer permanent roost sites. Satellite telemetry studies show that the bats are highly mobile, moving between Indonesia, Malaysia, Singapore and Thailand [33].

We found that flying foxes are consistently present throughout Perak State, including near the index farm, where we located two seasonal *P. vampyrus* roosts: one in Lenggong, Perak, <50 km from the index farm, where we observed a maximum population of approximately 2500 bats, and another near Tambun, Perak, within 2 km of the index farm, which had been previously reported in 1999 [32] and where we observed bats in 2003–2005 [33]. The distance between these roosts and the index farm is within the bats' nightly foraging range. Flying fox roosts were not found in the more densely populated pig-farming regions of southern Selangor and Negeri Sembilan [32,33] (figure 4).

#### 2.3.2. Bat serosurvey

Serological analysis demonstrates that evidence of NiV infection is ubiquitous throughout the distribution of flying fox populations in peninsular Malaysia (figure 4). Overall antibody prevalence in *P. vampyrus* and *P. hypomelanus* is 38.7 (*n* = 163) and 17.5 per cent (*n* = 80), respectively, consistent with previous reports of NiV antibodies or infection in flying fox populations from northern India and throughout southeast Asia [12,27–29]. Seropositive bats were found at all sampling sites, including the Lenggong colony near the index farm, which had a seroprevalence of 46 per cent (*n* = 28, June 2004).

## 3. Discussion

### 3.1. Process of emergence

**Figure 5.**
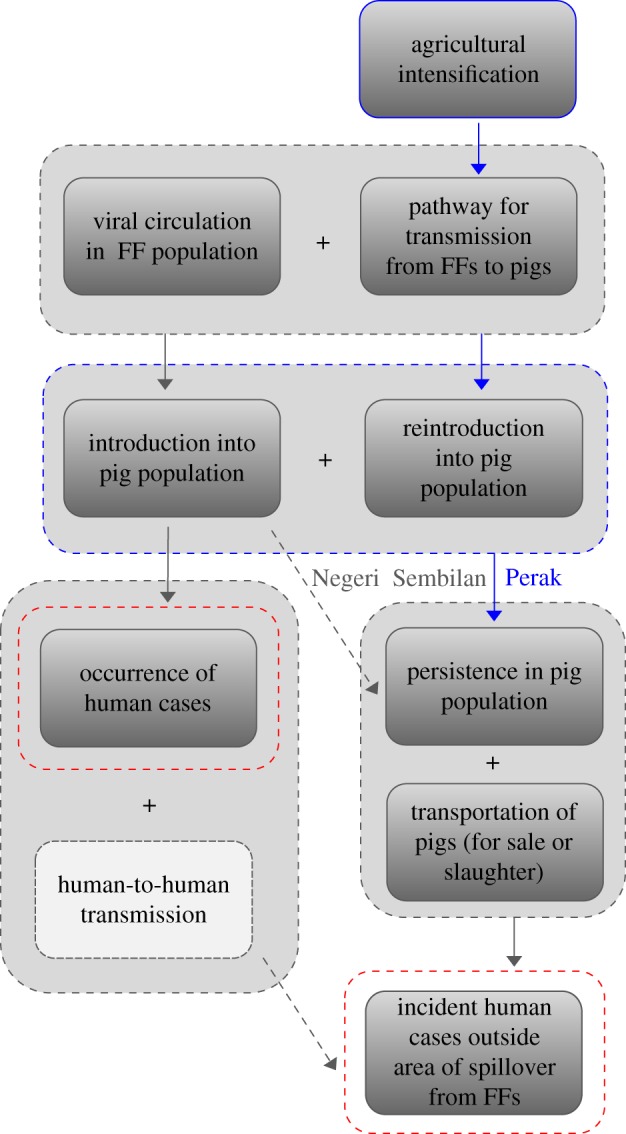
Complex causality of Nipah virus emergence in Malaysia. Each rectangle represents a component cause of emergence. Grey rectangles represent ‘realized’ component causes that were present in Malaysia, whereas white rectangles represent ‘potential’ component causes that did not occur. Boxes surrounding multiple component causes indicate complete or near-complete synergy between these causes. Arrows indicate that some component causes can bring about other component causes (creating a causal pathway), or can lead directly to an outcome of interest (phase I or phase II emergence, highlighted in red). We refer to component causes that brought about another component cause that had been a missing link in the causal pathway as ‘drivers’ (highlighted in dark blue). Alternative pathways that would have been sufficient to produce both phases of emergence—had they occurred—are indicated by dashed arrows. Specifically, introduction of Nipah virus into an area with extremely dense pig-farming activity (such as Port Dickson in Negeri Sembilan or Sabang Perai Utara in Pulau Penang) probably could have resulted in viral persistence without priming and reintroduction. However, these areas lacked factors further up the causal pathway: the absence of *Pteropus* bats in these areas prevented viral introduction prior to the establishment of an alternative source of infection (e.g. persistent viral circulation on the index farm in Kinta, Perak). Similarly, if Nipah virus in Malaysia had resulted in human-to-human transmission, an alternative causal pathway could have produced incident human cases outside the area of transmission from flying foxes, as was seen in the Faridpur outbreak in Bangladesh in 2004 [34], but there is no evidence that such human-to-human transmission occurred in Malaysia [35]. The causal pathway that was realized in Malaysia most probably involved (i) the creation of a pathway for transmission from bats to pigs via agricultural intensification (i.e. dual-use agriculture, or the practice of planting fruit trees on land used for livestock production, and increased fruit production through time) and (ii) repeated introduction of the virus into a high-turnover commercial pig population in Perak that led to viral persistence and set the stage for phase II of the emergence process. FF, flying fox.

NiV emergence in Malaysia occurred in two phases, and our current understanding of the processes leading to its emergence is outlined in figure 5. Phase I of emergence, the occurrence of human cases, required that only two criteria be met: circulation of the virus in a local bat population and the existence of a pathway for transmission from *Pteropus* bats to pigs. In phase I, early human cases were directly linked to the transmission of virus from bats to pigs, with all identified cases occurring in the immediate vicinity of the index farm where bat-to-pig transmission ignited rapid pig-to-pig transmission and produced a tight cluster of human cases on the index farm and an adjacent property.

Phase II of emergence, incident human cases outside the area of transmission from flying foxes, required that the criteria for phase I be met but also mandated the existence of a pathway for transmission to humans that had become unlinked from the wildlife reservoir. In principle, several scenarios could have produced such a pathway, as illustrated in figure 5; however, the evidence amassed here indicates that the causal pathway realized in the Malaysian outbreak involved priming for persistence and reintroduction on the index farm, which created a source of infection independent from the virus's circulation in flying foxes.

#### 3.1.1. Phase I emergence

The maturation of mango trees adjacent to pigsties on the index farm in 1987 created an opportunity for phase I emergence to occur, with transmission of the virus from flying foxes to pigs most likely to occur during fruiting or flowering. The electronic supplementary material includes a timeline of phase I emergence, which highlights the key events leading up to the introduction of NiV into the index farm. We propose that NiV did not emerge for some years after the maturation of the mango trees owing to chance events within the system, e.g. fruit bat population dynamics, migratory behaviour or the dynamics of NiV in bat populations. This is supported by our own work on Hendra virus which suggests that, when the virus is actively transmitted within a bat colony, there is a heightened chance of repeated spillover, but circulation of the virus in bats is unlikely at any given place and time [36]. On the other hand, we cannot rule out the possibility that specific local conditions in late 1996 and early to mid-1997 caused increased viral shedding among bats in the area surrounding the index farm, thereby increasing the risk of cross-species transmission to pigs.

Given the high mobility of flying foxes and the seasonal fluctuation in colony sizes in peninsular Malaysia [33], NiV is probably transmitted regularly between roost sites and throughout the region. Such dynamics explain the ubiquity of NiV antibodies, and our data suggest that flying foxes and possibly NiV were regularly present near the index farm in Perak prior to the 1998–1999 outbreak. Thus, we propose that it is plausible that flying foxes repeatedly introduced the virus onto the index farm in 1997.

#### 3.1.2. Phase II emergence

Our analyses have shown that the initial introduction on the index farm was most probably insufficient to induce persistent circulation of the virus. Furthermore, the lack of human cases on other farms between the time that this epizootic would have burned out and the reappearance of human cases associated with the index farm strongly suggests that the virus was not circulating on farms in the surrounding area in the interim. The short duration of this initial epizootic appears to have provided an insufficient window of time for transportation of pigs between farms to spread the infection. On the other hand, reintroduction of the virus into the ‘primed’ pig population on the index farm provided a substantially longer window of opportunity for spread. Our model's results indicate that reintroduction of the virus at the time of the reappearance of human cases on the index farm would have permitted enzootic circulation of the virus until the farm was culled in 1999.

During this time, the infection spread to other farms in the Tambun area, probably via movement of infected pigs from the index farm, which supplied gilts and piglets to smaller operations in the vicinity. Subsequent pig movement (including ‘fire sales’ of pigs in reaction to the cluster of human cases in September–November 1998) allowed the virus to spread throughout Perak and eventually south to Negeri Sembilan and Selangor [15]. Because there was no evidence of transmission between humans [35], the pattern of human infection necessarily followed the spread of the virus in pigs, explaining why human cases were much more widespread in 1998–1999 than in 1997. In addition, the high density of pig farms in many parts of the south (electronic supplementary material) may have allowed respiratory transmission of the virus between adjacent farms without physical movement of infected pigs, contributing to the rapid spread of infection and high number of human cases in the south. On the other hand, these areas were buffered from phase I emergence via bat-to-pig transmission by the absence of flying foxes in the area [33] (figure 4) and, possibly, a lesser degree of overlap between fruit and pig production (electronic supplementary material).

### 3.2. Drivers of emergence

We have identified two synergistic component causes [37] that precipitated each phase of emergence (figure 5). The existence of a pathway for transmission from flying foxes to pigs was a necessary complement to viral circulation in the bat population to produce phase I of emergence, and viral persistence in the pig population on the index farm created an infection source outside the flying fox reservoir that, combined with transportation of pigs, was sufficient to produce phase II of emergence. Each of these component causes was brought about by a phenomenon that—while not strictly necessary—did ultimately permit emergence in this context. We refer to these factors as ‘drivers’ of emergence, and table 1 outlines the evidence for interpreting each of these drivers as a causal factor. Agricultural intensification (namely, dual use of agricultural land and increases in production) resulted in the direct overlap of mango production and livestock rearing and therefore produced a pathway for a virus circulating in flying foxes to infect an intensively managed commercial pig population, driving phase I of emergence. Because NiV had a very low probability of persisting on the index farm without reintroduction (figure 3), priming for persistence was necessary to bring about enzootic circulation in this context and appears to have been sufficient to do so, driving phase II of emergence.

### 3.3. Broader implications

This study illustrates how a broad, interdisciplinary approach to the study of emerging zoonoses focused on data from specific emergence events can illuminate emergence processes. We have demonstrated the specific role of agricultural expansion, particularly dual use of agricultural land and intensified pig production practices, in NiV emergence. A role for long-term changes in species interactions has previously been hypothesized for numerous host-jumping viral species including various primate retroviruses [38,39], SARS [40] and avian influenza viruses, and our evidence suggests that cross-species transmission of NiV most probably resulted from the expanded interface between wild animal reservoirs and human or domestic animal populations, although it is possible that short-term ecological conditions were also a contributing factor.

Our findings have important implications for control of NiV in commercial pig farms, prevention of widespread epidemics in livestock and our general understanding of zoonotic disease emergence. First, although restrictions on planting fruit trees near pigsties appear to have prevented introduction of the virus into pig populations in Malaysia since 1999, there is a possibility of reintroduction both there and in other countries where flying foxes are found. Regular surveillance of pigs sent to markets and abattoirs in areas where pig farming overlaps flying fox distributions, along with hospital-based surveillance of encephalitis cases, may permit identification of initial NiV introductions and allow for early intervention and prevention of subsequent spread. Second, widespread prophylactic vaccination of pig populations is likely to be cost-prohibitive because of the rapid turnover of commercial pig populations. In addition, if vaccine coverage were not kept at a sufficient level, the dynamics of the virus when introduced into the population would resemble those following reintroduction on the index farm, inducing long-term persistence and increasing the risk of phase II emergence (electronic supplementary material).

Finally, an important feature of the NiV outbreak in Malaysia and Singapore was the two-phase process by which emergence occurred, which implies that there was a missed opportunity for earlier recognition of this novel aetiological agent and potential intervention. Zoonotic pathogens often go unnoticed during the initial stages of ‘viral chatter’—that is, repeated introductions that cause only a small number of cases [41]—because they are not yet easily transmissible in humans (e.g. simian retroviruses), are initially asymptomatic (e.g. HIV), are clinically similar to other diseases (e.g. NiV in Malaysia, which was originally misdiagnosed as Japanese encephalitis despite its epidemiological distinctiveness) and/or occur in areas with poor surveillance and diagnostic testing (e.g. NiV in Bangladesh and India). Other pathogens are noticed relatively quickly because they cause dramatic disease (e.g. Ebola haemorrhagic fever); however, even in these situations, we cannot rule out the possibility of previous undocumented cases, and the discovery of historical cases after the identification of an emerging pathogen is common (NiV [16], SARS [42], HIV [38] and H5N1 influenza [43]).

These patterns support previous calls for increasing targeted global surveillance and pathogen discovery in atypical disease outbreaks [44,45]. As the Malaysian NiV outbreak highlights, surveillance of livestock and livestock workers in regions of high wildlife biodiversity would improve the chances that viral chatter of wildlife origin is detected prior to a widespread epidemic in livestock or people. This approach may be critical for identifying new outbreaks of NiV, which has recently demonstrated a potential to produce propagated outbreaks in Bangladesh, where short chains of human-to-human transmission have occurred [34]. It may also provide a strategy for the identification of unknown henipaviruses, and other novel agents, prior to epidemic or pandemic emergence.

## 4. Material and methods

### 4.1. Agricultural production patterns

#### 4.1.1. Large-scale patterns

Malaysian mango production data (in metric tonnes per year) and domestic pig census data (in head of pig, censused at the end of the calendar year) for 1961–2005 were obtained from the Food and Agricultural Organization (FAO) of the United Nations' free online Agricultural Production database (FAOSTAT Classic; http://faostat.fao.org; accessed 15 November 2006). District-level density data for mango and pig production in 1997 were provided by the Malaysian government. Further details on these data are available in the electronic supplementary material.

#### 4.1.2. Patterns on the index farm

In order to better understand the history, layout and daily operation of the index farm, J.R.C.P. conducted field interviews of farm workers, including two private veterinarians who oversaw the health of the animals, and the orchard manager, as well as government veterinarians who oversaw and participated in the depopulation campaign.

### 4.2. Nipah virus on the index farm

#### 4.2.1. Dynamic models

Population dynamic models of production dynamics on the index farm were developed and parametrized from descriptions of farm-management practices provided during interviews with farm workers and detailed production records from 1 January 1995 to 31 October 1996. Models of infection dynamics were then constructed by building on these baseline population models. Dynamics were explored using a deterministic ordinary differential equation (ODE) model with a cut-off value of one infected individual (calculated as the sum of individuals in all exposed and infectious classes), below which the infection was considered to have gone extinct. A stochastic individual-based model (IBM) was then used to confirm that the qualitative dynamics observed in the ODE model were robust to the incorporation of empirically derived waiting time distributions and other factors that could not be represented in the ODE framework. The stochastic model was run for a wide range of parameter combinations to assess the probability of extinction following initial and subsequent introductions under different scenarios and to assess the range of transmission parameters consistent with observed seroprevalence levels in Breeding section 2. Detailed descriptions of the ODE model and the IBM are given in the electronic supplementary material.

#### 4.2.2. Data analysis

Statistical analyses of production records were developed to detect evidence of the virus in the breeding sections. A baseline model for pre-weaning piglet mortality was formulated for each breeding section on the basis of production records from 1 January 1995 through 31 October 1996, and candidate models were compared by Akaike information criterion. For all litters born after this period, a piglet mortality index (PMI) was calculated that indicates the extent to which the observed mortality deviates from the expected mortality for a litter of the same size during the baseline period. Litter-level data are shown in the electronic supplementary material.

For the sake of interpreting large-scale patterns over time, litters were binned according to farrowing date. We established a baseline for variation across bins in the average PMI based on the period from 1 January 1995 to 31 October 1996. In figure 1*b*, the binned PMI values are scaled in terms of the binned PMI percentile (relative to variation within the parametrization period). Absolute values and further details of the analysis are shown in the electronic supplementary material.

### 4.3. Bat surveys

#### 4.3.1. Bat census techniques

We collaborated with the federal wildlife department and enlisted a network of sport hunters as informants to locate and monitor flying fox roost locations throughout peninsular Malaysia. A full description of census techniques (and additional findings) has been published elsewhere [33]. We also developed two indices of flying fox density based on different expectations for how hunting practices reflect bat distribution. These indices and their underlying assumptions are described in the electronic supplementary material.

#### 4.3.2. Bat serosurvey

Flying foxes were non-randomly sampled using mist nets and anaesthetized. Three millilitres of blood was collected from the brachial vein and stored for 24 h at 4°C to allow for serum separation. The separated serum was then stored at −20°C until use. All bats were released at the site of capture after recovery from anaesthesia. Serum neutralization tests were conducted on all serum samples at the Australian Animal Health Laboratory, Geelong, Australia, under Biosafety Level 4 conditions. Details on anaesthesia and testing protocols are given in the electronic supplementary material.
